# Structure of a membrane-attack complex/perforin (MACPF) family protein from the human gut symbiont *Bacteroides thetaiotaomicron*

**DOI:** 10.1107/S1744309110023055

**Published:** 2010-07-31

**Authors:** Qingping Xu, Polat Abdubek, Tamara Astakhova, Herbert L. Axelrod, Constantina Bakolitsa, Xiaohui Cai, Dennis Carlton, Connie Chen, Hsiu-Ju Chiu, Thomas Clayton, Debanu Das, Marc C. Deller, Lian Duan, Kyle Ellrott, Carol L. Farr, Julie Feuerhelm, Joanna C. Grant, Anna Grzechnik, Gye Won Han, Lukasz Jaroszewski, Kevin K. Jin, Heath E. Klock, Mark W. Knuth, Piotr Kozbial, S. Sri Krishna, Abhinav Kumar, Winnie W. Lam, David Marciano, Mitchell D. Miller, Andrew T. Morse, Edward Nigoghossian, Amanda Nopakun, Linda Okach, Christina Puckett, Ron Reyes, Henry J. Tien, Christine B. Trame, Henry van den Bedem, Dana Weekes, Tiffany Wooten, Andrew Yeh, Jiadong Zhou, Keith O. Hodgson, John Wooley, Marc-André Elsliger, Ashley M. Deacon, Adam Godzik, Scott A. Lesley, Ian A. Wilson

**Affiliations:** a Stanford Synchrotron Radiation Lightsource, SLAC National Accelerator Laboratory, Menlo Park, CA, USA; b Joint Center for Structural Genomics, http://www.jcsg.org, USA; c Protein Sciences Department, Genomics Institute of the Novartis Research Foundation, San Diego, CA, USA; d Center for Research in Biological Systems, University of California, San Diego, La Jolla, CA, USA; e Program on Bioinformatics and Systems Biology, Sanford–Burnham Medical Research Institute, La Jolla, CA, USA; f Department of Molecular Biology, The Scripps Research Institute, La Jolla, CA, USA; g Photon Science, SLAC National Accelerator Laboratory, Menlo Park, CA, USA

**Keywords:** MACPF, membrane-attack complexes, perforins, transmembrane pores, pathogenesis

## Abstract

Membrane-attack complex/perforin (MACPF) proteins are transmembrane pore-forming proteins that are important in both human immunity and the virulence of pathogens. Bacterial MACPFs are found in diverse bacterial species, including most human gut-associated *Bacteroides* species. The crystal structure of a bacterial MACPF-domain-containing protein BT_3439 (Bth-MACPF) from *B. thetaiotaomicron*, a predominant member of the mammalian intestinal microbiota, has been determined. Bth-MACPF contains a membrane-attack complex/perforin (MACPF) domain and two novel C-terminal domains that resemble ribonuclease H and interleukin 8, respectively. The entire protein adopts a flat crescent shape, characteristic of other MACPF proteins, that may be important for oligomerization. This Bth-MACPF structure provides new features and insights not observed in two previous MACPF structures. Genomic context analysis infers that Bth-MACPF may be involved in a novel protein-transport or nutrient-uptake system, suggesting an important role for these MACPF proteins, which were likely to have been inherited from eukaryotes *via* horizontal gene transfer, in the adaptation of commensal bacteria to the host environment.

## Abstract 2

The crystal structure of a novel MACPF protein, which may play a role in the adaptation of commensal bacteria to host environments in the human gut, was determined and analyzed.

## 1. Introduction

Perforin (PF) and components of the membrane-attack complex (MAC; complement proteins C6–C9) are pore-forming proteins of the complement part of the innate immune system. They share a common domain (MACPF) that is also widely distributed in bacteria and protozoa, including many pathogens (Rosado *et al.*, 2008 ▶; Voskoboinik *et al.*, 2006 ▶). Perforin-like proteins in pathogens play an important role in virulence, for example, by disrupting the plasma membrane and facilitating parasite exit from host cells (Kafsack *et al.*, 2009 ▶). The recent structures of two MACPF proteins, a bacterial protein from *Photorhabdus luminescens* (Plu-MACPF) and the human complement membrane-attack complex component C8α, revealed an unexpected structural similarity to the well studied cholesterol-dependent cytolysins (CDCs) of many Gram-positive bacteria, thus suggesting a common mechanism of pore formation (Hadders *et al.*, 2007 ▶; Rosado *et al.*, 2007 ▶) by CDC and MACPF. CDCs form doughnut-shaped pores by the self-polymerization of 30–50 monomers on target membrane surfaces, followed by a major structural re­arrangement and the insertion of two helical regions (Tweten, 2005 ▶).

The Gram-negative anaerobic *Bacteroides thetaiotaomicron*, which is a predominant member of the human intestinal tract microbiota, is an important bacterium for the study of the symbiotic relationship between bacteria and humans (Xu *et al.*, 2003 ▶; Hooper & Gordon, 2001 ▶). Extracellular proteins are crucial for these functions in *B. thetaiotaomicron* and other gut microbes. We initiated a structural genomics project that aims to determine the structures of proteins that are unique to the secretome of human gut microbiota in order to provide broad insights into the molecular mechanisms of bacteria–host symbiosis and pathogenesis. We have selected proteins that do not display significant similarities to proteins of known structure and have determined the structures of more than 60 secreted human gut bacteria proteins thus far. Our structures have revealed that many of these proteins are distant homologs of well known protein families, which, in many cases, are undetectable based on sequence alone using even the most sensitive fold-detection algorithms. For example, the structure of a putative fimbriae assembly protein BT_1062 from *B. thetaiota­omicron* (PDB code 3gf8) revealed a fold similar to pili components of other bacteria despite no detectable sequential similarity (Xu *et al.*, 2010 ▶). Similarly, the structure of BVU_2987 (PDB code 3due) from *B. vulgatus* uncovered an unexpected similarity in fold to the β-­lactamase inhibitor protein (BLIP; Das *et al.*, 2010 ▶). Therefore, these proteins are also good candidates for exploring the evolution and divergence of protein structures and the underlying sequence–structure relationships. Here, we report the crystal structure of the MACPF protein BT_3439 from *B. thetaiotaomicron* (hereafter referred to as Bth-MACPF) at 2.46 Å resolution, which to our knowledge is the first structure of a potential CDC-like toxin from a gut symbiont.

## 2. Materials and methods

### 2.1. Protein production and crystallization

Clones were generated using the Polymerase Incomplete Primer Extension (PIPE) cloning method (Klock *et al.*, 2008 ▶). The gene encoding Bth-MACPF (GenBank NP_812351; Swiss-Prot Q8A267) was amplified by polymerase chain reaction (PCR) from *B. theta­iotaomicron* VPI-5482 genomic DNA using *PfuTurbo* DNA polymerase (Stratagene) and I-PIPE (Insert) primers (forward primer, 5′-­ctgtacttccagggcAATGAGGAGGAAACTAATAATTATACTC-3′; reverse primer, 5′-ctgtacttccagggcAATGAGGAGGAAACTAATA­ATTATACTC-3′; target sequence in upper case) that included sequences for the predicted 5′ and 3′ ends. The expression vector pSpeedET, which encodes an amino-terminal tobacco etch virus (TEV) protease-cleavable expression and purification tag (MGS­DKIHHHHHHENLYFQ/G), was PCR-amplified with V-PIPE (Vector) primers (forward primer, 5′-taacgcgacttaattaactcgtttaaacgg­tctccagc-3′; reverse primer, 5′-gccctggaagtacaggttttcgtgatgatgatgatg­atg-3′). The V-PIPE and I-PIPE PCR products were mixed to anneal the amplified DNA fragments together. *Escherichia coli* GeneHogs (Invitrogen) competent cells were transformed with the I-PIPE/V-­PIPE mixture and dispensed onto selective LB–agar plates. The cloning junctions were confirmed by DNA sequencing. Using the PIPE method, the gene segment encoding residues Met1–Thr18 was excluded from the final construct as it was predicted to encode a signal peptide. Expression was performed in selenomethionine-containing medium at 310 K. Selenomethionine was incorporated *via* inhibition of methionine biosynthesis (Van Duyne *et al.*, 1993 ▶), which does not require a methionine-auxotrophic strain. At the end of fermentation, lysozyme was added to the culture to a final concentration of 250 µg ml^−1^ and the cells were harvested and frozen. After one freeze–thaw cycle, the cells were sonicated in lysis buffer [50 m*M* HEPES pH 8.0, 50 m*M* NaCl, 10 m*M* imidazole, 1 m*M* tris(2-carboxyethyl)phosphine–HCl (TCEP)] and the lysate was clarified by centrifugation at 32 500*g* for 30 min. The soluble fraction was passed over nickel-chelating resin (GE Healthcare) pre-equilibrated with lysis buffer, the resin was washed with wash buffer [50 m*M* HEPES pH 8.0, 300 m*M* NaCl, 40 m*M* imidazole, 10%(*v*/*v*) glycerol, 1 m*M* TCEP] and the protein was eluted with elution buffer [20 m*M* HEPES pH 8.0, 300 m*M* imidazole, 10%(*v*/*v*) glycerol, 1 m*M* TCEP]. The eluate was buffer-exchanged with TEV buffer (20 m*M* HEPES pH 8.0, 200 m*M* NaCl, 40 m*M* imidazole, 1 m*M* TCEP) using a PD-10 column (GE Healthcare) and incubated with 1 mg TEV protease per 15 mg of eluted protein. The protease-treated eluate was run over nickel-chelating resin (GE Healthcare) pre-equilibrated with HEPES crystallization buffer (20 m*M* HEPES pH 8.0, 200 m*M* NaCl, 40 m*M* imidazole, 1 m*M* TCEP) and the resin was washed with the same buffer. The flowthrough and wash fractions were combined and concentrated to 19.8 mg ml^−1^ by centrifugal ultrafiltration (Millipore) for crystallization trials. Bth-MACPF was crystallized by mixing 100 nl protein solution with 100 nl crystallization solution in a sitting drop over a 50 µl reservoir volume using the nanodroplet vapor-diffusion method (Santarsiero *et al.*, 2002 ▶) with standard JCSG crystallization protocols (Lesley *et al.*, 2002 ▶). The crystallization reagent consisted of 5%(*v*/*v*) 2-methyl-2,4-pentanediol, 12%(*v*/*v*) polyethylene glycol 6000, 0.1 *M* HEPES pH 6.7. A cube-shaped crystal of approximate dimensions 40 × 40 × 30 µm was harvested after 42 d at 277 K for data collection. Glycerol was diluted to 10%(*v*/*v*) using the reservoir solution and then added to the drop in a 1:1 ratio as a cryoprotectant prior to mounting. Initial screening for diffraction was carried out using the Stanford Automated Mounting system (SAM; Cohen *et al.*, 2002 ▶) at the Stanford Synchrotron Radiation Lightsource (SSRL, Menlo Park, California, USA).

The oligomeric state of Bth-MACPF in solution was determined using a 1 × 30 cm Superdex 200 size-exclusion column (GE Healthcare) coupled with miniDAWN static light-scattering (SEC/SLS) and Optilab differential refractive-index detectors (Wyatt Technology). The mobile phase consisted of 20 m*M* Tris–HCl pH 8.0, 150 m*M* NaCl and 0.02%(*w*/*v*) sodium azide. The molecular weight was calculated using *ASTRA* v.5.1.5 software (Wyatt Technology).

### 2.2. Data collection, structure solution, refinement and analysis

Multi-wavelength anomalous diffraction (MAD) data were collected on beamline 9-2 at the SSRL at wavelengths corresponding to the peak (λ_1_), high-energy remote (λ_2_) and inflection (λ_3_) wavelengths of a selenium MAD experiment (see Table 1 ▶). The data sets were collected at 100 K using a MAR CCD 325 detector. The MAD data were integrated and reduced using *XDS* and scaled with the program *XSCALE* (Kabsch, 2010 ▶). Selenium sites were located with *SHELXD* (Sheldrick, 2008 ▶) and refined using *autoSHARP* (mean figure of merit of 0.34 with 22 selenium sites; Bricogne *et al.*, 2003 ▶). Density modification was performed by *SOLOMON* (Abrahams & Leslie, 1996 ▶) and automatic model building was performed by *Buccaneer* (Cowtan, 2006 ▶). Iterative model building and refinement were performed with *Coot* (Emsley & Cowtan, 2004 ▶) and *REFMAC* (Winn *et al.*, 2003 ▶), respectively. The refinement included experimental phase restraints in the form of Hendrickson–Lattman coefficients and TLS refinement with four TLS groups per chain (residues 36–56, 66–389, 390–493 and 494–558). *CCP*4 programs were used for data conversion and other calculations (Collaborative Computational Project, Number 4, 1994 ▶). Data-processing and refinement statistics are summarized in Table 1 ▶. The quality of the crystal structure was evaluated using *MolProbity* (Chen *et al.*, 2010 ▶) and *WHAT IF* (Vriend, 1990 ▶). *HHpredict* was used for protein-homology detection and function prediction (Soding *et al.*, 2005 ▶). Signal peptides were analyzed using *SignalP* (Emanuelsson *et al.*, 2007 ▶) and *LipoP* (Juncker *et al.*, 2003 ▶). Oligomers of Bth-MACPF with *C*_16_ symmetry were predicted using *SymmDOCK* (Schneidman-Duhovny *et al.*, 2005 ▶). Molecular graphics were prepared with *PyMOL* (DeLano Scientific). Sequence alignments were rendered using *TEXshade* (Beitz, 2000 ▶).

## 3. Results and discussion

### 3.1. Bioinformatics analysis

MACPF domains are widely distributed in eukaryotes, but are sporadic in bacteria. Only ∼40 bacterial MACPF proteins are cataloged in the PFAM database (PF01823; Bateman *et al.*, 2004 ▶). Chlamydiaceae contain 13 closely related MACPF proteins (Ponting, 1999 ▶). *Bacteroides* contain about a third of all bacterial MACPF proteins. The others are found in diverse bacterial species from proteobacteria, actinomycetales and cyanobacteria. It has been suggested that these proteins were acquired from eukaryotes through horizontal gene transfer in order to adapt to the intracellular environment of the host (Ponting, 1999 ▶; Wolf *et al.*, 1999 ▶). Preliminary phylogenetic analysis (data not shown) suggests that the Bacteroidetes branch is likely to represent an independent horizontal gene-transfer event. Thus, MACPFs in the human gut microbiome may play an important role in the symbiotic relationship, but their specific functions are currently unknown.

**Figure 1:**
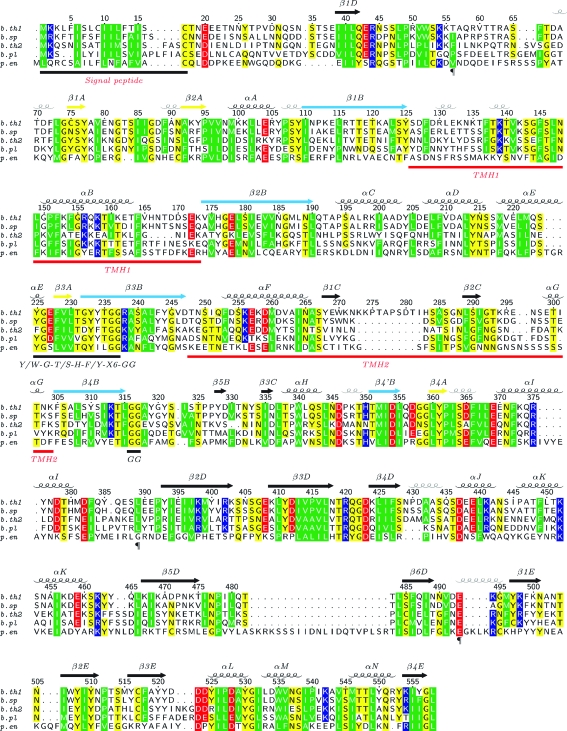
Multiple sequence alignment of Bth-MACPF homologs (sequence identity <90%) with the same domain architecture. The sequence numbering and secondary-structure elements of Bth-MACPF are shown at the top and domain boundaries (¶) and sequence motifs are shown at the bottom. Charged residues are highlighted in red (negative) and blue (positive), hydrophobic residues in green and hydrophilic residues in yellow. The following sequences are shown: *b.th1*, *B. thetaiotaomicron* BT_3439 (Bth-MACPF); *b.sp*, *Bacteroides* sp. 2_2_4 (UniProt accession C3QVE5); *b.th2*, *B. thetaiotaomicron* BT_3437; *b.pl*, *B. plebeius* DSM 17135 (UniProt accession B5CX96); *p.en*, *P. endodontalis* ATCC 35406 (UniProt accession C3J7W9).

Bacterial MACPFs are highly divergent in sequence and domain architecture. Homologs that have significant similarity over the entire sequence of Bth-MACPF are found mostly in other human-related Bacteroidetes, including unclassified *Bacteroides* sp. (strains 2_1_22, 2_2_4 and D1), *B. fragilis* 3_1_12 (Bfra3_17507), *B. plebeius* DSM 17135 (BACPLE_01336), *B. intestinalis* DSM 17393 (BACINT_00423) and *Porphyromonas endodontalis* ATCC 35406 (POREN0001_1212) (Fig. 1 ▶), but also in the recently sequenced deep-sea *Zunongwangia profunda* SM-A87 (ZPR_2061). MACPFs from *Bacteroides* are unique as most of them contain lipoprotein signal peptides (Juncker *et al.*, 2003 ▶) that are not present in other bacterial MACPFs. *B. thetaiotaomicron* contains two homologous MACPFs (BT_3439 and BT_3437; 33% sequence identity) that are likely to form part of an operon (see more detailed discussion below), as well as a third more distant paralog (BT_3120) that consists of only an MACPF domain. *B. fragilis* YCH46 (BF1566, BF1634 and BF2685) and *B. intestinalis* DSM 17393 (BACINT_00423, BACINT_00829 and BACINT_03190) each contain three MACPFs, with only one protein in each species having the same domain architecture as Bth-MACPF.

**Figure 2:**
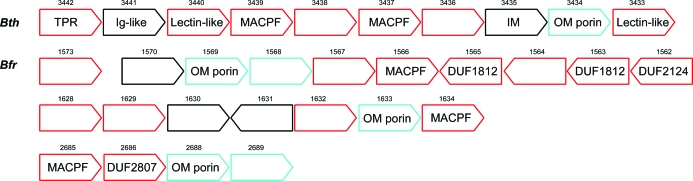
Genomic context of MACPF proteins in two completed genomes of *Bacteroides*: *B. thetaiotaomicron* (Bth) and *B. fragilis* YCH46 (Bfr). Predicted lipoproteins are shown as red boxes. Proteins containing other signal peptides are colored cyan. The locus number of each gene is shown at the top. IM, inner membrane protein; OM, outer membrane protein; DUF, domain of unknown function; TPR, protein containing tetratricopeptide repeats.

Bth-MACPF is located among a cluster of uncharacterized proteins (BT_3442 to BT_3433) that form a putative operon and which are located directly downstream of a well defined operon of cell-division and cell-wall biosynthesis proteins such as FtsZ, FtsA, FtsQ and MurC. This cluster, which appears to contain internal duplications resulting in three homologous pairs (BT_3436/BT_3438, BT_3437/BT_3439 and BT_3433/BT_3440), is rich in potential pore-forming proteins (BT_3433, BT_3434, BT_3437, BT_3439 and BT_3440). Most of the proteins in the cluster also contain similar lipoprotein signal peptides (Fig. 2 ▶), suggesting that they are localized to a common area in the cell. BT_3433 and BT_3440 are likely to have a trefoil fold resembling that of hemolytic pore-forming lectins (Mancheno *et al.*, 2005 ▶). BT_3434 is likely to be an outer membrane porin, while BT_3435 is a putative inner membrane protein with three transmembrane helices. BT_3441 is a homolog of a hypothetical protein BVU_0276 from *B. vulgatus*, the structure of which has also been determined by the JCSG (PDB code 3d33). It has an immunoglobulin-like fold that is common in cell-surface proteins such as fibronectin and complement C3. BT_3442 is a multi-domain protein containing TPR motifs, which often mediate protein interaction. Therefore, Bth-MACPF is associated with several pore-forming proteins, suggesting a possible role in a cross-membrane transport system. The association of *Bacteroides* MACPFs with lipoproteins and outer membrane porins is also observed in *B. fragilis* YCH46 (Fig. 2 ▶).

Bth-MACPF was predicted to be an extracellular protein by *PSORTb* (Gardy *et al.*, 2005 ▶) and *SOSUI*_*GramN*_ (Imai *et al.*, 2008 ▶). The N-terminal region of Bth-MACPF (^1^MKKLFISLCIILFTISC^17^) matches the lipoprotein signal peptide pattern of Gram-negative bacteria, which usually consists of one or more positive charged residues followed by a stretch of hydrophobic residues and a lipobox motif L(A/S)(G/A)C (Hayashi & Wu, 1990 ▶). Similar lipoprotein signal peptides are also present in structural subunits of the major and minor fimbriae FimA and Mfa1 of *P. gingivalis*, which is a close phylogenetic relative of *B. thetaiotaomicron*, suggesting a common mechanism of translocation across the membrane (Shoji *et al.*, 2004 ▶). Lipoproteins are transported across the inner membrane by the general secretion pathway. On the periplasmic face of the inner membrane, the invariant cysteine residue is modified by the diacylglyceryl transferase (Lgt), followed by cleavage of the peptide before the diacylglyceride cysteine by signal peptidase II (LspA) and further modification of the diacylglyceride cysteine by aminoacyl transferase (Lnt; Tokuda, 2009 ▶). These proteins are then sorted to their final destinations, but the details of the final steps of translocation of extracellular lipoproteins in *Bacteroides* are currently not clear. The final products could either be tethered to the outer membrane or cleaved and released to the extracellular medium and may be dependent on other residues in close proximity to the cysteine (*e.g.* the conserved acidic residue at position +4; Fig. 1 ▶).

### 3.2. Structural determination

The *BT_3439* gene of *B. thetaiotaomicron* encodes a predicted lipoprotein with a molecular weight of 63 425 Da (residues 1–558) and a calculated isoelectric point of 5.5. We determined the structure using the high-throughput pipeline of the Joint Center for Structural Genomics (JCSG; Lesley *et al.*, 2002 ▶) as part of the National Institute of General Medical Sciences’ Protein Structure Initiative (PSI; http://www.nigms.nih.gov/Initiatives/PSI/). A selenomethionine derivative of Bth-MACPF was expressed in *E. coli* with an N-terminal TEV-cleavable His tag and was purified by metal-affinity chromatography. To improve the likelihood of obtaining crystals, the predicted N-­terminal signal peptide (residues 1–18) was not included in the clone construct. The data were indexed in the orthorhombic space group *P*2_1_2_1_2_1_ and the structure was determined at 2.46 Å resolution with two molecules per asymmetric unit using the MAD method. The structure was refined to a final *R* factor of 20.9% and an *R*_free_ of 25.2%. The model of Bth-MACPF displays good geometry, with an all-atom clash score of 7.8, and the Ramachandran plot produced by *MolProbity* (Chen *et al.*, 2010 ▶) shows that all residues are in allowed regions, with 96.7% in favored regions. The final model of Bth-MACPF contains residues *A*/*B*36–558, 239 waters and other solvent molecules that were present in the crystallization or cryoprotection reagents, including one MPD [(4*S*)-2-methyl-2,4-pentanediol] molecule, one chloride ion and three ethylene glycol molecules. The residual residue (Gly0) from the cleaved N-terminal purification tag and segments *A*/*B*19–35, *A*/*B*57–65, *A*277–286, *B*272–286 and *A*482–483 were not included in the model owing to a lack of interpretable electron density. Additionally, side chains for 17 residues were only partially modeled owing to disorder. Data-collection, refinement and model statistics are summarized in Table 1 ▶.

### 3.3. Overall structure

**Figure 3:**
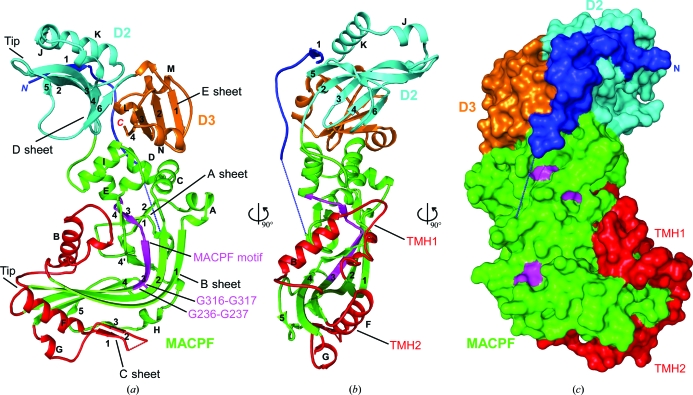
Crystal structure of Bth-MACPF. (*a* and *b*) Ribbon representations of Bth-MACPF in orthogonal views. The color scheme is as follows: the extended N-terminal region is shown in blue, the MACPF domain in shown in green with TMHs in red and the MACPF motif in magenta, domain D2 is shown in cyan and domain D3 is shown in orange. The β-sheets (A–E) and helices (A–N) are labeled alphabetically as in Fig. 1 ▶; 3_10_-helices are not labeled. (*c*) Surface representation of Bth-MACPF color coded by domain as in (*a* and *b*).

Bth-MACPF (Fig. 3 ▶) adopts a flat crescent shape with molecular dimensions of 93 × 58 × 44 Å. The two monomers in the asymmetric unit are nearly identical (with an overall r.m.s.d. of 0.67 Å for 493 C^α^ atoms) with larger deviations located at the two tips, mostly owing to a slight opening of the crescent in molecule *B* compared with molecule *A*. Bth-MACPF consists of three structured domains: an MACPF domain (residues 66–389) and two C-terminal domains, D2 (residues 390–492) and D3 (residues 493–558) (Figs. 3*a* and 3*b* ▶). Residues 36–56 of the N-terminus of Bth-MACPF adopt an extended conformation and pack against parts of the MACPF (residues 388–392), D2 (residues 449–474) and D3 (residues 523–531) domains with a total buried surface area of 2029 Å^2^ (Fig. 3 ▶ *b*). The interface contains 32 hydrogen bonds and helps to maintain overall structural integrity. This arrangement places the predicted N-terminal membrane-attachment site (Cys18) away from the MACPF domain. The remaining N-­terminal residues that were included in the construct (residues 19–35 and 57–65) were not observed in the electron density and are most likely to be flexible in solution. Furthermore, Bth-MACPF is likely to be a monomer in solution, as supported by crystal-packing analysis and analytical size-exclusion chromatography (data not shown).

The MACPF domain contains two four-stranded β-sheets (A and B) in the central core, which is decorated by several helical insertions. The A sheet with its short strands (strand order 2134) and the B sheet with long strands (strand order 1234) assemble to form a twisted S shape. The B sheet itself is very distorted and bends fairly abruptly in the middle by ∼90°. This arrangement of central β-sheets with characteristic geometry is common to both MACPFs and CDCs and allowed the classification of MACPF and CDC into a single family (Rosado *et al.*, 2007 ▶; Hadders *et al.*, 2007 ▶). The last strand of the B sheet is interrupted (strands 4 and 4′) by an insertion (residues 316–350) at the bend of the sheet. Insertions between β1–β2 and β3–β4 (TMH1 and TMH2, respectively) correspond to the so-called TMH regions of CDCs, which unfold and form transmembrane β-hairpins. TMH1 (residues 126–173) contains one helix (αB) and two short 3_10_-­helices that pack against the inner surface of the B sheet. TMH2 (residues 248–304) contains an antiparallel αβ–βα structure that sits on the outer surface of the B sheet. The two strands in TMH2 and another strand from the 4–4′ (B-sheet) insertion forms another β-­sheet (C sheet) parallel to the B sheet. The MACPF motif Y/W-G-T/S-H-F/Y-*X*_6_-GG (Ponting, 1999 ▶; Rosado *et al.*, 2007 ▶) is located on strands 3A and 3B (Fig. 3 ▶ *a*). The corresponding Bth-MACPF region (^225^YG**EFV** *X*_6_GG^237^) is more divergent from the consensus, with nonconserved changes at positions 3–5. Two glycines from the MACPF motif (Gly236 and Gly237) and two additional nearby conserved glycines (Gly316 and Gly317; Figs. 1 ▶ and 3 ▶) are likely to be essential for structural flexibility in MACPF and CDC (Rosado *et al.*, 2007 ▶).

The A sheet is crowned by four helices: αI and a three-helix insertion (αC–αE) between β2B (β2 of the B sheet) and β3A. These helices form the interface between the MACPF and D2/D3 domains. Both D2 and D3 are layered structures with a central β-sheet pro­tected by helices on two sides (see below). The D2 and MACPF interface involves interaction between αD and αI of MACPF and the β3–β4 and β5–β6 loops of D2 and buries a surface area of ∼1000 Å^2^ (500 Å^2^ each). The interface is mostly hydrophilic. In particular, a buried Asp423 in D2 forms a bifurcated hydrogen bond to Arg375 of MACPF. D3 functions as a wedge between D2 and MACPF, with a similar interface area on either side (total ∼1400 Å^2^ for D3). Leu558 is buried with its C-terminal carboxyl group forming a hydrogen-bond network involving the conserved residues Arg420 and Tyr530. Additionally, the interaction between domains is further stabilized by the N-­terminal extended region (residues 36–56) described above. Gap-volume indices between these interacting components are less than 1.7, which is consistent with the expected average (1.8) for intrachain domain–domain interfaces (Jones *et al.*, 2000 ▶). Thus, we conclude that the domain arrangement observed in the crystal structure is likely to be representative of the functional protein and not a crystallization artifact.

### 3.4. D2 and D3 domains

**Figure 4:**
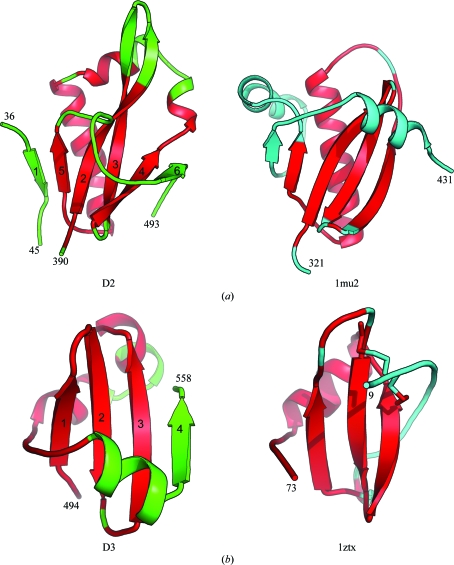
Structural comparisons of the D2 and D3 domains. (*a*) Structural comparison between D2 and the connector domain of HIV reverse transcriptase (PDB code 1mu2). (*b*) Structural comparison between D3 and a viral chemokine (PDB code 1zxt). Equivalent C^α^ atoms are shown in red.

The MACPF domain is usually attached to other auxiliary domains that are expected to regulate the function of MACPF. As discussed earlier, both C-terminal domains of Bth-MACPF are only detected in its closest homologs in sequence-similarity searches (Fig. 1 ▶). The D2 and D3 domains show some structural similarity: both have an α/β fold with βββαβ topology. However, most structural comparison programs fail to recognize this similarity and also fail to identify significant similarities to other proteins. The βββαβ core of D2 and D3 can be partly matched to other structures (Fig. 4 ▶), for instance to proteins with the YegP-like fold (SCOP ID 160112), which is characterized by an internal repeat of two domains with a βββαβ core. Other examples include the connector domain (residues 321–431; PDB code 1mu2; Ren *et al.*, 2002 ▶) of HIV reverse transcriptase (*Z* = 3.6; r.m.s.d. 3.3 Å for 68 aligned C^α^ atoms; sequence identity 6%), which is likely to have evolved from the ribonuclease H domain (Malik & Eickbush, 2001 ▶; Fig. 4 ▶ *a*). However, the C-terminal portions of the two structures differ significantly. Domain D3 is similar, for instance, to a viral chemokine (PDB code 1zxt; Luz *et al.*, 2005 ▶), with an r.m.s.d. of 2.2 Å (sequence identity 5%) for 44 C^α^ atoms (Fig. 4 ▶ *b*). Chemokines adopt a βββα interleukin 8-like structure stabilized by two conserved disulfide bonds. D3 lacks the long cysteine-containing N-terminal portion observed in chemokines. Instead, it contains an αβ C-terminal extension and forms a βββαααβ overall structure. The βββαβ motif is most likely to represent a repeated structural unit that can be found in nonhomologous proteins with different functions, thus limiting the interpretation of structural similarity in terms of common function.

### 3.5. Homology of MACPF domains

The MACPF domain in Bth-MACPF is homologous to human MACPFs, as indicated by the significant sequence similarity recognized, for instance, by *FFAS* (Jaroszewski *et al.*, 2005 ▶) and *HHpredict* (Hildebrand *et al.*, 2009 ▶) and by three-dimensional structural similarity using the *DALI* server (Holm & Sander, 1995 ▶). The first two *DALI* hits are the only two previously determined MACPF structures: Plu-MACPF (PDB code 2qp2; Rosado *et al.*, 2007 ▶) and the C8α MACPF domain (PDB codes 2qqh and 2rd7; Hadders *et al.*, 2007 ▶; Slade *et al.*, 2008 ▶). Bth-MACPF is most similar to Plu-MACPF, with a *Z* score of 17.4, which corresponds to an r.m.s.d. of 3.8 Å and 16% sequence identity for 247 aligned C^α^ atoms. The second hit, human C8α (PDB code 2qqh), can be superimposed onto Bth-MACPF with 218 aligned C^α^ atoms, an r.m.s.d. of 5.0 Å and 14% sequence identity (*Z* = 12.3). More distant similarity is also apparent between Bth-MACPF and CDCs, such as the thiol-activated cytolysin perfringolysin O (PFO; PDB code 1m3i; Rossjohn *et al.*, 1997 ▶; *Z* = 7.2, r.m.s.d. 5.2 Å and 11% sequence identity for 198 aligned C^α^ atoms). The structural similarity between MACPF domains and the CDC family of toxins has previously been noted, which led to the proposal that MACPF domains use a CDC-like mechanism for pore formation (Rosado *et al.*, 2007 ▶; Hadders *et al.*, 2007 ▶). In this model, TMH1 and TMH2 undergo conformational changes to form antiparallel hairpins so that the extended β-sheet can oligomerize through the open edges of β1 and β4.

**Figure 5:**
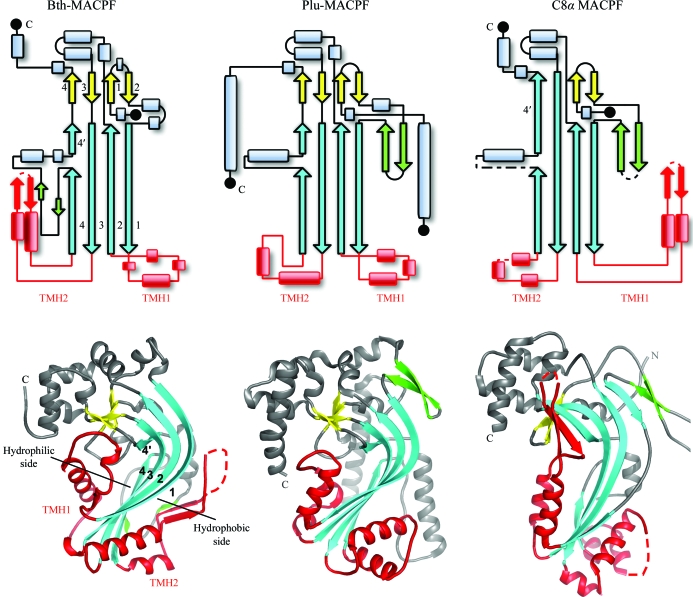
Structural comparison of the MACPF domains in Bth-MACPF, Plu-MACPF and C8α. Top: comparison of the secondary-structure topology diagrams of MACPF domains (sheet A, yellow; sheet B, cyan; TMHs, red). Bottom: ribbon representation of MACPF proteins in the same orientation and color coded as in the topology diagrams.

The similarity between the three MACPF domains is even more significant at the topological level (Fig. 5 ▶). All contain a common core consisting of sheet A and sheet B. Various insertions occur at specific locations in the conserved strands, most notably between β2A and β1B, β4B and β4B′, β1B and β2B (TMH1), β3B and β4B (TMH2) and β2B and β3A. One common helix within the β4B–β4B′ insertion (αH of Bth-MACPF) is conserved in all known MACPFs and harbors several highly conserved residues (*e.g.* Trp340) that interact with the region containing the critical glycines that were discussed above. The β4B–β4B′ insertion in Bth-MACPF contains two additional short strands that augment the B sheet and the C sheet, respectively. As a result, this insertion in Bth-MACPF is more similar to PFO. The additional short β-strand in the B sheet of CDCs (β5B in Bth-MACPF) prevents premature oligomerization by blocking access to β4 (Ramachandran *et al.*, 2004 ▶). The β-hairpin insertion between β2A and β1B of the C8α MACPF domain and Plu-MACPF are replaced by one helix (αA) and a 3_10_-helix in Bth-MACPF. This region of C8α is involved in the interaction with the C8γ subunit (Slade *et al.*, 2008 ▶). The TMH regions of MACPFs and CDCs are generally not conserved in sequence (Rosado *et al.*, 2007 ▶). TMHs of Bth-MACPF contain short stretches of amphipathic regions which might be important for forming transmembrane hairpins (Fig. 1 ▶). Both TMHs of Bth-MACPF (48 and 57 amino acids) are longer than the TMHs of CDCs, which generally consist of ∼30 amino acids. Longer TMH regions (∼60 amino acids) are also observed in C8α, C9 and perforin and are likely to be a general feature of MACPF. C8α and Bth-MACPF both contain an αβ–βα hairpin, but in different locations (TMH1 in C8α and TMH2 in Bth-MACPF). Interestingly, the two faces of the B sheet in all three MACPFs display amphipathic properties. The interface between the B sheet and TMH1 is mostly polar, whereas the TMH2 interface is more tightly packed and hydrophobic (Fig. 5 ▶).

### 3.6. Functional implications

The helical insertion between β2B and β3A is involved in docking the D2 and D3 domains to the Bth-MACPF domain. These helices are also present in Plu-MACPF and C8α MACPF, but are currently not implicated in protein–protein interactions. Both Plu-MACPF and Bth-MACPF contain additional C-terminal domains. However, the locations of these domains are completely different. The C-terminal β-prism domain of Plu-MACPF is located on the opposite side of the central core (left corner of lower figure of Plu-MACPF in Fig. 5 ▶) compared with D2 and D3 (upper left corner) in Bth-MACPF. The arrangements of these auxiliary domains may reflect their different roles. The β-prism domain of Plu-MACPF is similarly located compared with domain 4 of PFO and may interact with the membrane directly (Rosado *et al.*, 2007 ▶). In contrast, D2 and D3 of Bth-MACPF, which are distant from the TMH regions, seem more likely to play a role in protein–protein interaction (*e.g.* polymerization or interaction with BT_3442) rather than membrane attachment. The shape of Bth-MACPF appears to be self-complementary, which could facilitate ring-like self-assembly (Hadders *et al.*, 2007 ▶) to form pores across membranes. Modeling studies suggest that it is feasible for Bth-MACPF to polymerize *via* the C-terminal auxiliary domains. A model with 16 copies of Bth-MACPF forms a doughnut-shaped molecule with an inner radius of 110 Å, similar in pore size to the the C9 MACPF model (Hadders *et al.*, 2007 ▶). The multimer interface involves docking a helical wedge from D2 and D3 (helices K, L and M) into the D2–MACPF interface (D sheet and helix I). The formation of protein complexes involving Bth-MACPF may facilitate structural changes in the MACPF domain which are necessary to form the porin-like transmembrane pore.

MACPFs are well known for killing cells by forming pores and thus are potential virulence factors. Here, we demonstrate the existence of a novel subfamily of secreted MACPF proteins in commensal bacteria. Unfortunately, the physiological functions of these proteins are currently unknown. The properties of the MACPF/CDC fold, such as structural flexibility and membrane penetration, may be utilized for nonlytic purposes (Rosado *et al.*, 2007 ▶) and Bth-MACPF may be involved in novel protein-secretion or nutrient-uptake systems. Alternatively, MACPFs may protect the bacteria from host immunity through molecular mimicry (Stebbins & Galan, 2001 ▶; Kohm *et al.*, 2003 ▶). For example, the presence of these molecules on the cell surface may prevent the assembly of the host MACPF complex. Another possibility is that MACPFs may function as potential toxins, such as bacteriocins against Gram-positive bacteria. Bacteriocins are often produced by nonpathogenic bacteria that colonize the human body and may help to prevent infection by opportunistic pathogenic bacteria. Furthermore, it remains possible that these bacterial MACPFs are virulence factors towards the host under certain conditions, as gut symbionts, such as *B. fragilis*, are also opportunistic pathogens. It is well documented that many bacterial virulence-factor genes are located within genomic islands (Juhas *et al.*, 2009 ▶). The clustering of potential pore-forming outer-membrane toxins in the *B. thetaiota­omicron* genome suggest that this region could be a pathogenicity island acquired through horizontal gene transfer, as predicted by a genome-wide genomic islands study (Ho Sui *et al.*, 2009 ▶).

Although the functions of the MACPFs represented by Bth-MACPF remain to be elucidated, our study provided clues that they are important targets for further exploration of how symbiotic microbes adapt to and influence their host environments. Additional information about the proteins described in this study is available from TOPSAN (Krishna *et al.*, 2010 ▶) at http://www.topsan.org/explore?PDBid=3kk7.

## Supplementary Material

PDB reference: MACPF family protein, 3kk7

## Figures and Tables

**Table 1:** Summary of crystal parameters, data-collection and refinement statistics for Bth-MACPF (PDB code 3kk7) Values in parentheses are for the highest resolution shell.

	λ_1_ MADSe	λ_2_ MADSe	λ_3_ MADSe
Space group	*P*2_1_2_1_2_1_		
Unit-cell parameters (Å)	*a* = 78.4, *b* = 127.2, *c* = 138.3		
Data collection			
Wavelength (Å)	0.9791	0.9184	0.9792
Resolution range (Å)	49.4–2.46 (2.59–2.46)	48.0–2.80 (2.95–2.80)	48.0–2.80 (2.95–2.80)
No. of observations	342564	122453	121355
No. of reflections	49779	34098	33698
Completeness (%)	97.3 (94.2)	98.2 (98.2)	97.0 (99.7)
Mean *I*/σ(*I*)	10.6 (2.3)	7.3 (2.0)	8.6 (2.6)
*R*_merge_ on *I*†	0.123 (0.75)	0.153 (0.69)	0.125 (0.52)
Model and refinement statistics			
Resolution range (Å)	49.4–2.46		
No. of reflections (total)	49764		
No. of reflections (test)	2514		
Completeness (%)	97.3		
Data set used in refinement	λ_1_ MADSe		
Cutoff criterion	|*F*| > 0		
*R*_cryst_‡	0.209		
*R*_free_§	0.252		
Stereochemical parameters			
Restraints (r.m.s.d. observed)			
Bond lengths (Å)	0.014		
Bond angles (°)	1.47		
Average isotropic *B* value (Å^2^)	40.2¶		
ESU†† based on *R*_free_ (Å)	0.27		
Protein residues/atoms	1001/8046		
Solvent molecules	244		

† *R*_merge_ = 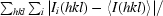 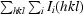.

‡ *R*_cryst_ = 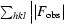 − 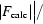 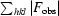, where *F*_calc_ and *F*_obs_ are the calculated and observed structure-factor amplitudes, respectively.

§ *R*_free_ is the same as *R*_cryst_ but for 5% of the total reflections chosen at random and omitted from refinement.

¶ This value represents the total *B* that includes TLS and residual *B* components.

†† Estimated standard uncertainty in coordinates (Collaborative Computational Project, Number 4, 1994 ▶; Cruickshank, 1999 ▶).
